# Analysis of pulmonary vascular injury and repair during *Pseudomonas aeruginosa* infection-induced pneumonia and acute respiratory distress syndrome

**DOI:** 10.1177/2045894019826941

**Published:** 2019-02-22

**Authors:** Ashley S. Lindsey, Lydia M. Sullivan, Nicole A. Housley, Anna Koloteva, Judy A. King, Jonathon P. Audia, Diego F. Alvarez

**Affiliations:** 1 Department of Physiology and Cell Biology, University of South Alabama, Alabama, USA; 2 Department of Microbiology and Immunology, University of South Alabama, Alabama, USA; 3 Center for Lung Biology, University of South Alabama, Alabama, USA; 4 Department of Pathology and Translational Pathobiology, Louisiana State University Health, Louisiana, USA; 5 University of South Alabama College of Medicine, Alabama, USA

**Keywords:** *Pseudomonas aeruginosa*, pulmonary endothelial cells, endothelial permeability, pulmonary edema, acute respiratory distress syndrome (ARDS)

## Abstract

Herein we describe lung vascular injury and repair using a rodent model of *Pseudomonas aeruginosa* pneumonia-induced acute respiratory distress syndrome (ARDS) during: 1) the exudative phase (48-hour survivors) and 2) the reparative/fibro-proliferative phase (1-week survivors). Pneumonia was induced by intratracheal instillation of *P. aeruginosa* strain PA103, and lung morphology and pulmonary vascular function were determined subsequently. Pulmonary vascular function was assessed in mechanically ventilated animals in vivo (air dead space, P_a_O_2_, and lung mechanics) and lung permeability was determined in isolated perfused lungs ex vivo (vascular filtration coefficient and extravascular lung water). At 48 hours post infection, histological analyses demonstrated capillary endothelial disruption, diffuse alveolar damage, perivascular cuffs, and neutrophil influx into lung parenchyma. Infected animals displayed clinical hallmarks of ARDS, including increased vascular permeability, increased dead space, impaired gas exchange, and decreased lung compliance. Overall, the animal infection model recapitulated the morphological and functional changes typically observed in lungs from patients during the exudative phase of ARDS. At 1 week post infection, there was lung histological and pulmonary vascular functional evidence of repair when compared with 48 hours post infection; however, some parameters were still impaired when compared with uninfected controls. Importantly, lungs displayed increased fibrosis and cellular hyperplasia reminiscent of lungs from patients during the fibro-proliferative phase of ARDS. Control, sham inoculated animals showed normal lung histology and function. These data represent the first comprehensive assessment of lung pathophysiology during the exudative and reparative/fibro-proliferative phases of *P. aeruginosa* pneumonia-induced ARDS, and position this pre-clinical model for use in interventional studies aimed at advancing clinical care.

## Introduction

A distinguishing characteristic of the pulmonary circulation is its potential for exposure to environmental stressors originating from the air we breathe and/or from the circulation. Pulmonary circulation dysfunction typically occurs in cases where sterile or infectious stressors perturb alveolar epithelial and endothelial cell homeostasis, leading to loss of barrier and transport properties.^1–4^ When accompanied by dysregulated inflammatory responses,^5^ pulmonary circulation compromise can culminate in ARDS, a critical care condition with a mortality rate of 40–60%.^6^ ARDS is clinically defined as respiratory distress not fully driven by cardiac failure or fluid overload^7^ but due to disruption of the epithelial and alveolar–capillary barriers, increased extravascular edema, and severe alterations in gas exchange and lung mechanics.^1,3,4,8,9^ Extravascular edema is a compounding factor that physically disrupts lung function via inactivation of surfactant, causing alveolar collapse upon expiration and increasing tissue resistance to airflow.^10^ In addition, perivascular edema further increases tissue resistance and decreases lung compliance,^11^ typified by a loss of lung distensibility and increased work of breathing.^12,13^ Compromise of the pulmonary capillaries increases physiological dead space, areas of the lung that are ventilated but not perfused, and increases pulmonary shunting, areas of the lung that are perfused but not ventilated.^1,3,9,14^ This ventilation/perfusion (V/Q) mismatch impairs gas exchange, leading to decreased arterial oxygen tension (P_a_O_2_).^14–16^ As the injury worsens or progresses towards resolution, cellular hyperplasia, microthrombi formation within the vasculature, and fibrinoid deposits within the matrix define the reparative/fibro-proliferative phase of ARDS.^3,4,9^ Considering the vast array of underlying etiologies and the extremely complex nature of the host response to ARDS, therapeutic advances aimed at treating this clinical condition will rely on development of comprehensively characterized disease models.

The opportunistic Gram-negative pathogen *Pseudomonas aeruginosa* is a predominant cause of nosocomial pneumonia in critically ill patients, which often progresses to ARDS.^17,18^ Upon inoculation into the airway, *P. aeruginosa* infects alveolar epithelial cells and resident macrophages, eliciting release of pro-inflammatory cytokines that recruit immune cells into the lung parenchyma and airspaces.^19–21^ The combined effects of infection and inflammation exacerbate barrier damage, allowing direct infection of, and damage to, the pulmonary vascular endothelium.^2,22–24^ Systemic dissemination of the pathogen (via the disrupted barrier or through pulmonary lymphatics^25^) along with attendant endotoxemia and cytokine storm can precipitate the onset of sepsis, leading to further pulmonary vascular endothelial barrier disruption.^19,26,27^ The heterogeneous nature of infection caused by different *P. aeruginosa* isolates is attributable to variability in their cadre of virulence factors. Highly pathogenic isolates express a type III secretion system (T3SS), a molecular syringe that delivers exoenzyme (Exo) effectors directly into the cytoplasm of an infected eukaryotic host cell.^18,21,22,28,29^ The four known *P. aeruginosa* Exo effectors are 1) ExoU, a potent phospholipase A_2_ cytotoxin; 2) ExoS, a bifunctional Rho GTPase-activating protein and ADP-ribosyltransferase with cytotoxic activity; 3) ExoT, a second bifunctional Rho GTPase-activating protein and ADP-ribosyltransferase; and 4) ExoY, a promiscuous nucleotidyl cyclase. Interestingly, the Exo effectors are inactive in the bacterial cytoplasm and become activated upon T3SS-mediated delivery into the host cell cytoplasm due to interaction with their cognate eukaryotic co-factor(s).^30–32^ *P. aeruginosa* isolates vary in the combination of Exo effectors expressed, and the incidence of ARDS and subsequent patient mortality is high among individuals infected with isolates expressing a functional T3SS along with the ExoU effector.^21,33,34^ Importantly, *P. aeruginosa* is ranked second on the World Health Organization Critical Priority 1 list of highly antibiotic-resistant pathogens. The ESKAPE pathogens (*Enterococcus faecium*, *Staphylococcus aureus*, *Klebsiella pneumoniae*, *Acinetobacter baumannii*, *P. aeruginosa*, and *Enterobacter* species) are recognized as major causes of nosocomial infections, with growing emphasis on identifying novel therapies to treat these highly antibiotic-resistant pathogens.^35^

Our previous studies determined that *P. aeruginosa*-induced pneumonia in rats progresses to bacteremia and sepsis resulting in multiple organ system dysfunction, highlighted by evidence of liver and kidney dysfunction.^19^ In this previous study we compared a wild type *P. aeruginosa* strain with a mutant strain lacking a functional T3SS and showed that mutation of the T3SS significantly attenuated virulence. Even addition of a 1 log higher dose of the T3SS mutant strain failed to elicit injury comparable to the wild type strain. Together, these data suggest that bacterial virulence factors, rather than endotoxin, are primary drivers of injury in this model. However, the lung vascular pathophysiological progression of injury and repair during infection with a well-characterized wild type *P. aeruginosa* strain has not yet been assessed. Based on the prevalence of *P. aeruginosa* as a nosocomially acquired cause of pneumonia-induced ARDS, the need for robust laboratory models to elucidate syndrome progression and identify novel interventional strategies is patent. Thus, the goal of the present study was to comprehensively describe the exudative and reparative/fibro-proliferative phases in a rodent model of ARDS using *P. aeruginosa* strain PA103 expressing a functional T3SS that secretes the highly cytotoxic ExoU effector and ExoT. Direct intratracheal instillation of PA103 reproducibly established infection of the lower airways, elicited inflammatory cell infiltration, and disrupted epithelial and endothelial cell barriers at 48 hours post inoculation. Airway and interstitial edema was accompanied by diffuse alveolar damage and the hallmark impairments of lung function described in ARDS patients. At 1 week post inoculation, animals displayed recovery of lung function, and histological analyses revealed the presence of fibrinoid deposits and alveolar remodeling reminiscent of findings in patients succumbing to ARDS.

## Methods

### Ethics statement

This study was performed in accordance with the Guide for the Care and Use of Laboratory Animals of the National Institutes of Health.^36^ The experimental protocol was approved by the University of South Alabama Institutional Animal Care and Use Committee (IACUC). Animals were provided with food and water *ad libitum*. All surgeries were performed after confirming that animals had achieved anesthetic plane, and all efforts were made to minimize suffering. Post inoculation with *P. aeruginosa*, animals were humanely euthanized when they exhibited signs of high respiratory distress or by direct recommendation of the veterinarian. For all other studies, control and infected animals were humanely euthanized at 24 hours, 48 hours, or 1 week post inoculation. Animals were euthanized with a barbiturate overdose followed by exsanguination, a procedure conforming to recommendations from the veterinarian panel of euthanasia and the IACUC.

### Bacterial culture conditions and inoculum preparation

*P. aeruginosa* strain PA103 was used in the studies described herein. PA103 is a laboratory-domesticated clinical isolate that is non-motile due to lack of flagella but expresses a functional T3SS and secretes the exoenzyme effectors ExoU and ExoT.^34^ Bacteria were routinely cultured overnight at 37℃ in culture dishes containing the minimal E salts medium of Vogel and Bonner and 1.5% agar (Bacto Difco). Bacteria were aseptically scraped into 1 mL sterile saline (0.9%), suspended by vortex mixing, and collected by centrifugation (8000 × *g*, 8 min, ambient temperature). Post centrifugation, the supernatant was discarded, 1 mL sterile saline was added, and the pellet was suspended by vortex mixing. This stock solution was subsequently diluted with sterile saline to give the desired final optical density at 600 nm (OD_600_) corresponding to the infectious dose to be instilled in a volume of 200 µL. As a reference, an OD_600_ of 0.03 (measured on a Thermo Spectronic Helios Gamma spectrophotometer) corresponds to 5 × 10^7^ colony forming units (CFUs)/200 µL, as determined by performing dilution series and direct plate counting. The diluted inoculation solution was held at ambient temperature for no longer than 1 hour prior to use in an experiment.

### Animal inoculation procedure

Adult male CD rats (250–325 g body weight) were anesthetized via intraperitoneal (I.P.) injection with a ketamine–xylazine mixture (75 and 5 mg/kg, respectively). Upon confirmation of anesthetic plane (toe pinch reflex), the animal was placed into Fowler’s position (75^o^ angle), the trachea was exposed by incision and retraction of surrounding muscle, and 1 × 10^6^, 5 × 10^7^, 1 × 10^8^, or 1 × 10^9^ CFUs of PA103 in 200 µL of saline was instilled into the trachea (directly caudal to the thyroid gland) by injection through a 27-gauge needle. Control animals were instilled with 200 µL of sterile saline alone. Animals were returned to their cages and monitored during recovery from anesthesia. Post-recovery animals were provided with food and water *ad libitum*. For the remainder of the experiments described, a dose of 5 × 10^7^ was used.

### Microscopy

At either 48 hours or 1 week post inoculation, animals were anesthetized via I.P. injection of sodium pentobarbital (50 mg/kg). After confirmation of anesthetic plane, a tracheal tube was inserted and secured with a ligature, and mechanical ventilation was initiated at 7 mL/kg tidal volume; 55 breaths/minute respiratory rate; 3 cmH_2_O positive end-expiratory pressure (PEEP); and 75% inspiratory:expiratory ratio. A thoracotomy was performed and digital images were taken. The lungs were tied off at full tidal volume (peak of inspiration), excised, and prepared for the different microscopic approaches. For light microscopy, lungs were immersed in formalin and embedded in paraffin. From each paraffin-embedded lung specimen, two 5 µm sections were cut and placed on glass slides, the next three 5 µm sections were cut and wasted, and the next two 5 µm sections were placed on glass slides. For each animal, two lung sections from different planes were stained with either hematoxylin and eosin (H&E) or Trichrome stain. This procedure ensured sampling of two distinct alveolar fields. For each H&E-stained tissue section, images from five random fields of view were captured on an EOS-Nikon light microscope coupled to a CCD camera at 4× and 20× magnification. For each experimental condition (i.e., control cohort, 48-hour cohort, and 1-week cohort; *n* = 3 animals per cohort), at least 120 individual images were counted to determine intra-acinar fluid volume fractions using a point-counting strategy.^1,37^ A random sampling of the images were counted by a second, blinded observer in order to control for any potential bias. The intra-acinar spaces that contained fluid were quantified by point counting, which involves placing a 200 by 200 grid over each 20× image, and all intersections of the grid lines on an intra-acinar area that contained fluid were counted. Areas that contained fluid were represented as a fraction of the total grid intersections. Perivascular cuff frequency was determined by counting the number of vessels between 25 and 200 µm that had cuffs present as compared with the total number of pulmonary vessels, and the cuff volume area of these vessels was measured as a fraction of the total wall area.

An independent set of lung lobes was processed for transmission electron microscopy (TEM) to provide ultrastructural analyses of the lung parenchyma. Tissue was fixed in cacodylate buffer containing 3% glutaraldehyde (Electron Microscopy Sciences). Specimens were post-fixed with 1% osmium tetroxide (Electron Microscopy Sciences), dehydrated with a graded alcohol series, and embedded in PolyBed 812 resin (Polysciences). Semi-thin sections (1 µm) were cut and stained with Toluidine blue (Electron Microscopy Sciences) and were examined by light microscopy as described above. Thin sections (80 nm) were cut with a diamond knife, stained with uranyl acetate (Electron Microscopy Sciences), counterstained with Reynold’s lead citrate (Mallinckrodt), and examined by TEM using a Philips CM 100 (FEI).

### Animal ventilation and thoracotomy procedure

In a separate cohort, at either 48 hours or 1 week post inoculation, animals were anesthetized via I.P. injection of sodium pentobarbital (50 mg/kg). After confirmation of anesthetic plane, the animal was placed into Fowler’s position (75^o^ angle), and the carotid artery and trachea were surgically exposed. A heparinized catheter was placed into the right carotid artery and secured with a ligature in order to measure physiological parameters. A tracheal tube was inserted and secured with a ligature, and mechanical ventilation was initiated using a SCIREQ Flexivent 5.1 set to 7 mL/kg tidal volume; 55 breaths/minute respiratory rate; 3 cmH_2_O PEEP; and 75% inspiratory:expiratory ratio. Respiratory pressures were monitored. Pancuronium bromide was given intravenously (I.V.) as a bolus at 0.1 mg/kg to achieve paralysis and allow lung mechanics measurements. Exhaled air was bubbled into water, and blood was collected from the carotid artery. Water and blood samples were used to measure exhaled gases and arterial gases, respectively.

At the terminus of lung mechanics measurements, animals were transferred to a Harvard Apparatus Inspira Advanced Safety Ventilator and the ventilator settings maintained as above. To prepare lungs for measurement of the filtration coefficient (K_f_) using a gravimetric approach,^19,38^ a thoracotomy was performed and catheters were secured in the pulmonary artery and left atrium. The heart and lungs were isolated *en bloc* and suspended from a force transducer. Lungs were ventilated with an O_2_/CO_2_ mixture (21% / 5%) and constantly perfused with 30% autologous blood in Earle’s buffer solution with 4% bovine serum albumin (for 1000 mL of Earle’s: 870 mL dH_2_O, 100 mL 10× Earle’s balanced salt solution, and 30 mL 7.5% NaHCO_3_ solution in dH_2_O). All chemicals were from Millipore Sigma. The perfusate flow was maintained at 0.04 mL/kg of body weight, pH was 7.4, and the temperature at the pulmonary artery root was maintained at 36℃. The K_f_ measurement was initiated by increasing the venous pressure to 15 cmH_2_O for 15 minutes and measured as the rate recorded over the last 2 minutes. K_f_ is expressed as the rate of lung weight gain divided by the corresponding change in capillary pressure (after raising venous pressure minus basal pressure).

### Physiologic measurements

The exhaled gas and arterial blood samples were used to calculate physiologic dead space (VD/VT) by measuring the arterial CO_2_ tension minus the CO_2_ tension in exhaled air over the arterial CO_2_ tension ((P_a_CO_2_ – P_E_CO_2_) ÷ P_a_CO_2_). Lung mechanics were measured using specific perturbations (Snapshot 90v5.2 and Prime 8v5.2) on the SCIREQ Flexivent 5.1 ventilator. Lung mechanics reported are 1) dynamic compliance (C), a measure of lung distensibility that assesses air flow-dependent lung elastic recoil; 2) total lung resistance (R), a measure of the resistance in small airways; 3) airway resistance (R_n_), a measure of resistance in the conducting airways; 4) tissue damping (G), a measure of the resistive forces of the lung parenchyma that assesses air flow-independent distal airway elastic recoil; and 5) tissue elastance (H), a measure of elastic forces of the lung parenchyma indicative of the force required to overcome the elastic recoil of the lung upon inspiration. Cardiac output and central venous pressure were measured by advancing Millar catheters into the left ventricle via the carotid artery and into the jugular vein, respectively. Data were recorded digitally using an Advanced Instrument transducer.

In an independent experimental cohort, extravascular lung water (EVLW) content was determined after measurement of lung mechanics. The EVLW was normalized based on amounts of hemoglobin in the tissue versus total hemoglobin in the blood (comparing wet with dry ratios) as previously described.^39^

### Enumeration of bacteria in the lung

In an independent cohort of animals, the left lung was aseptically excised and sectioned. The lower third of the sample was minced with scissors and dispersed into 600 µL saline solution using a tissue homogenizer. The entire sample was divided equally between six to eight Pseudomonas isolation agar (Bacto Difco) plates and incubated overnight at 37℃. Plates were counted the following day, and the CFUs are reported per gram of lung tissue.

### Collagen deposition assay

The remainder of the left lung sample described above (enumeration of bacteria in the lung) was prepared for analysis of hydroxyproline levels as an indicator of collagen deposition using a colorimetric assay. The Cell Biolabs Inc. Hydroxyproline Assay Kit was used as per the manufacturer’s instructions. Hydroxyproline levels are reported per gram of lung tissue.

### Statistical analysis

Data are expressed as mean ± SEM from at least seven independent experiments for physiology measurements (unless otherwise indicated) and from at least three independent experiments for microscopy measurements. Data were analyzed by one-way analysis of variance (ANOVA) followed by Newman–Keuls multiple comparison post hoc test when comparing three groups, or unpaired *t*-test when comparing two groups. Differences with *P* < 0.05 were considered significant. All analyses were performed using Prism v6.1 software (Graphpad, Inc.).

## Results

### Survival and morphological evidence of injury and repair during P. aeruginosa lung infection

**Fig. 1.**
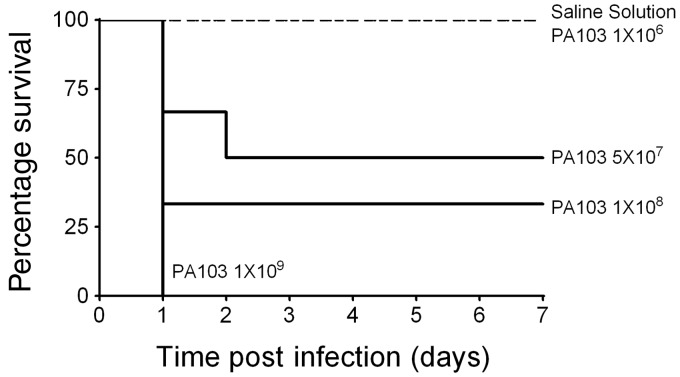
Instillation of wild type *P. aeruginosa* strain PA103 (expressing T3SS effectors ExoU and ExoT) into the airways of wild type CD rats produces a dose–response effect on survival. PA103 was suspended into sterile normal saline solution and inoculated at the doses indicated in the figure. Animals were followed for 1 week post inoculation. These studies determined an infectious dose of 5 × 10^7^ CFUs as the LD_50_, which was used in all subsequent experiments.

Our study rationale was based on the current lack of knowledge regarding the natural evolution of ARDS^4,9^ induced by ESKAPE pathogens,^35^ an emerging group of highly antibiotic-resistant microbes. In the present study we comprehensively describe the exudative (acute) and reparative/fibro-proliferative (late) phases of ARDS using a rodent model of *P. aeruginosa*-induced pneumonia. Infections were induced using *P. aeruginosa* strain PA103 expressing a functional T3SS that secretes the highly cytotoxic ExoU effector and the ExoT effector. We first determined the infectious dose necessary to cause disease in otherwise healthy adult male CD rats. Fig. 1 shows the dose–response effect on survival over a 1-week period following intratracheal instillation of *P. aeruginosa*. Instillation of saline as a control procedure had no adverse effects. To confirm the presence of *P. aeruginosa*, animals were randomly selected and lung homogenates plated onto Pseudomonas isolation agar. At 24 and 48 hours post inoculation, distinctive green colonies indicative of *P. aeruginosa* were observed. At 1 week post inoculation, fewer colonies were observed compared with 48 hours post inoculation (Fig. S1, available in the online Supplemental Material). Based on these data, an inoculation dose of 5 × 10^7^ CFUs per animal was used for all subsequent experiments.

*P. aeruginosa*-induced pneumonia is known to cause diffuse alveolar damage, increased pulmonary vascular permeability, and impaired gas exchange, which are identified as major clinical features of ARDS.^2–4,8,9,22–24^ Infected rat lungs were harvested at either 48 hours or 1 week post inoculation, and control lungs injected with saline solution were harvested at 1 week post inoculation. Macroscopically, control lungs had a homogeneous pink coloration with no signs of dark or bloody areas (Fig. S2(a)). However, infected lungs displayed patchy discoloration indicative of isolated areas of fluid and/or blood extravasation at ∼48 hours post inoculation (Fig. S2(b)). Overt signs of injury persisted at 1 week post inoculation with areas of patchy discoloration (Fig. S2(c)), but the damage appeared less substantial compared with lungs examined at 48 hours post inoculation. To account for heterogeneity in the host response to infection, which could bias interpretation of the observed diminished injury at 1 week post inoculation, we randomized animal selection for analyses at 48 hours versus 1 week.

**Fig. 2.**
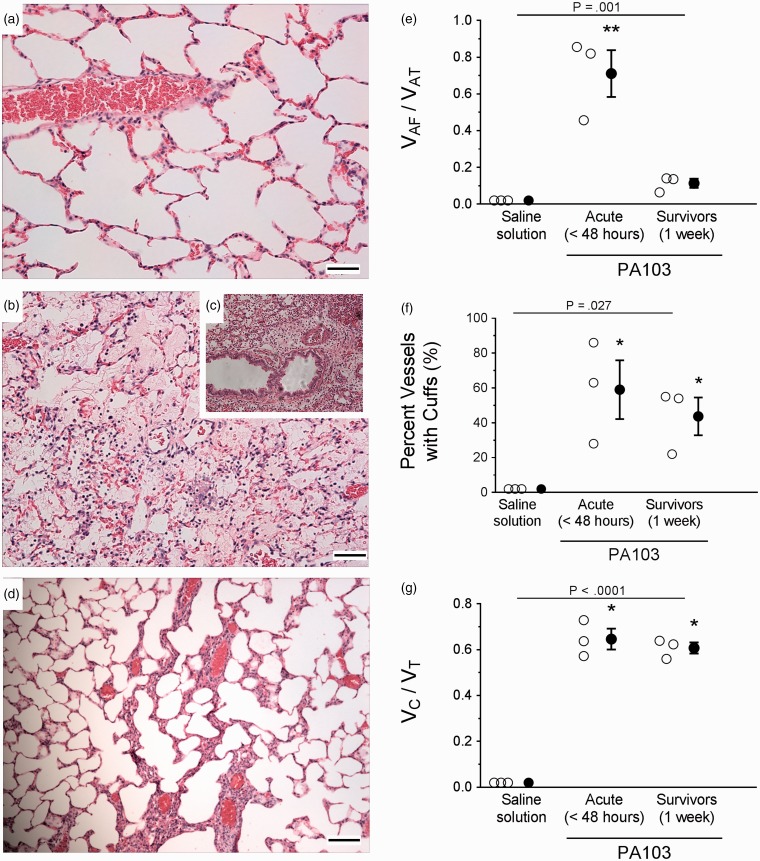
Microscopic evidence of the acute and fibro-proliferative phases of PA103-induced lung injury. Lungs were fixed and sectioned for H&E staining (panels (a) to (d), reference bar = 25 µm). (a) Lungs from control animals inoculated with saline solution alone were characterized by thin alveolar walls, clear alveolar spaces, and very few neutrophils, which were confined to the septal network. (b) Lungs from animals at 48 hours post PA103 inoculation were characterized by thickening of the alveolar walls, edema and hemorrhage, perivascular edema, accumulation of neutrophils within the alveolar airspaces, and vascular congestion. (c) Inset panel highlighting the presence of perivascular cuffing and cellular infiltrates. (d) Lungs from animals at 1 week post PA103 inoculation were characterized by alveolar wall thickening, with scant edema and hemorrhage, perivascular edema, or accumulation of neutrophils within the alveolar airspaces. (e) Quantitation of fluid accumulation in intra-acinar spaces. Airway instillation of PA103 increased the fraction of intra-acinar spaces that contained fluid as compared with the total intra-acinar spaces observed (V_AF_/V_AT_). **P = .001 when compared with all other groups by one-way ANOVA. (f) Quantification of vessels with perivascular cuffs. Airway instillation of PA103 increased the percentage of vessels with perivascular cuffs. *P = .027 compared with saline solution by one-way ANOVA. (g) Quantitation of fluid accumulation in perivascular spaces. Airway instillation of PA103 increased the fraction of perivascular cuff volume as compared with the total wall volume (vessel area plus cuff area, V_C_/V_T_). *P < .0001 compared with saline solution by one-way ANOVA. For panels (e) to (g), open circles represent measurements from individual lungs and closed circles indicate average ± SEM.

**Fig. 3.**
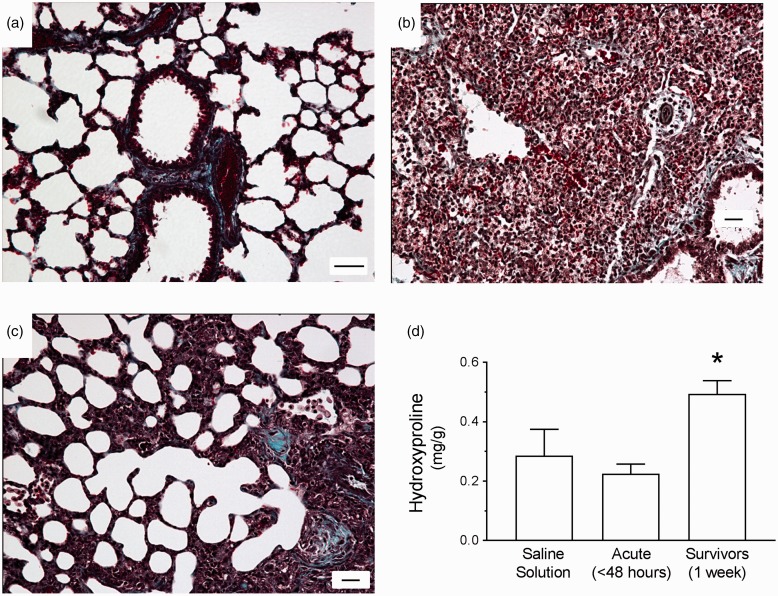
Evidence of extracellular matrix deposition and fibrotic lesions during PA103-induced lung injury. For panels (a) to (c), lungs were fixed and sectioned for trichrome staining, where blue staining represents regions of collagen deposition (reference bar = 20 µm). (a) Lungs from control animals inoculated with saline solution alone were characterized by thin alveolar walls and clear alveolar spaces. (b) Lungs from animals at 48 hours post PA103 inoculation were characterized by thickening of the alveolar walls, edema, increased cellular infiltrates, and evidence of extracellular matrix deposition and fibrotic lesions. (c) Lungs from animals at 1 week post PA103 inoculation were characterized by marked extracellular matrix deposition and dense fibrotic lesions indicative of injury and repair. (d) Assessment of hydroxyproline levels as a marker of collagen deposition. Lungs from animals at 1 week post PA103 inoculation displayed significantly higher levels of hydroxyproline compared with lungs from animals at 48 hours post infection. *P < .02 compared with acute by one-way ANOVA. Bars represent average ± SEM.

Histological evidence of *P. aeruginosa*-induced vascular injury was observed by light microscopy of lung sections. Fixed lung specimens were sectioned and stained with H&E (Fig. 2(a) to (d)), or semi-thin sections were stained with Toluidine blue (Fig. S3(a) to (f)). Control lungs were characterized by 1) continuous thin alveolar walls and capillary vessels; 2) clear alveolar airspaces; 3) clear delineation of the three layers of extra-alveolar vessels with no evident patent lymphatics in the adventitia; and 4) few neutrophils confined to the septal network (Fig. 2(a) and S3(a)). In contrast, lung sections prepared 48 hours post inoculation with PA103 were characterized by 1) thickened alveolar walls with disruption of capillary vessels and presence of microthrombi (Fig. 2(b), S3(b), and S3(d)); 2) evident fluid accumulation in airspaces with areas of atelectasis and consolidation, and hemorrhage (Fig. 2(b), S3(b), and S3(d)); 3) cuffs surrounding extra-alveolar vessels with patent lymphatics in the adventitia (Fig. 2(c) and S3(c)); and 4) increased inflammatory infiltrates in airspaces, interstitium, and perivascular cuffs (Fig. 2(b), 2(c), S3(b), S3(c), and S3(e)). Consolidation of the alveolar spaces with inflammatory infiltrates is definitive of pneumonia, and our findings are reminiscent of diffuse alveolar damage.^9^ Lungs at 1 week post inoculation with PA103 were characterized by 1) thin alveolar walls (Fig. 2(d)); 2) scant evidence of fluid accumulation in airspaces, atelectasis, or hemorrhage (Fig. 2(d)); 3) persistence of perivascular cuffs (Fig. S3(f)); and 4) few neutrophils within the alveolar airspaces, interstitium, or perivascular cuffs (Fig. 2(d)). Overall, histological evidence after 1 week post PA103 inoculation was suggestive of injury resolution and repair as compared with injured lungs harvested at 48 hours post inoculation.

The extent of endothelial barrier damage observed by microscopy and H&E staining was quantified by measuring intra-acinar fluid volume fractions, the percentage of pulmonary vessels with perivascular cuffs, and the perivascular cuff volume fraction. Lungs from animals at 48 hours post PA103 inoculation displayed a significantly increased fraction of intra-acinar spaces containing fluid when compared with control animals or 1-week infected animals (Fig. 2(e)). Lungs at 1 week post inoculation were not different compared with control lungs (Fig. 2(e)). The frequency of perivascular cuffing was significantly higher at 48 hours and 1 week post inoculation compared with control lungs (Fig. 2(f)). There were no differences between 48-hour and 1-week infection groups (Fig. 2(f)). A similar trend was observed for the perivascular cuff volume fraction (Fig. 2(g)). The fact that less fluid was present within the airspaces of 1-week infected animals compared with 48-hour infected animals suggests repair and fluid clearance. Interestingly, the frequency of perivascular cuffing and the cuff volume fractions remained elevated in the 1-week infection group, suggesting that the effects of infection on permeability and/or the ability to repair differ between pulmonary vascular segments.

We further assessed damage and potential for repair by evaluating lungs stained with trichrome, which identifies fibrotic and collagen deposits. Fig. 3(a) and (b) showed that compared with controls, lungs harvested at 48 hours post inoculation displayed increased deposition of extracellular matrix and presence of fibrotic foci. At 1 week post inoculation, damage was less evident, but there were regions with increased extracellular matrix deposition suggestive of tissue remodeling (Fig. 3(c)). Moreover, we assayed lung homogenates for levels of hydroxyproline, a marker of collagen deposition. Fig. 3(d) showed that at 1 week post inoculation, hydroxyproline levels were higher than controls and significantly higher than 48-hour lungs. Together, these data demonstrate that tissue remodeling occurs in the setting of PA103-induced pneumonia.

**Fig. 4.**
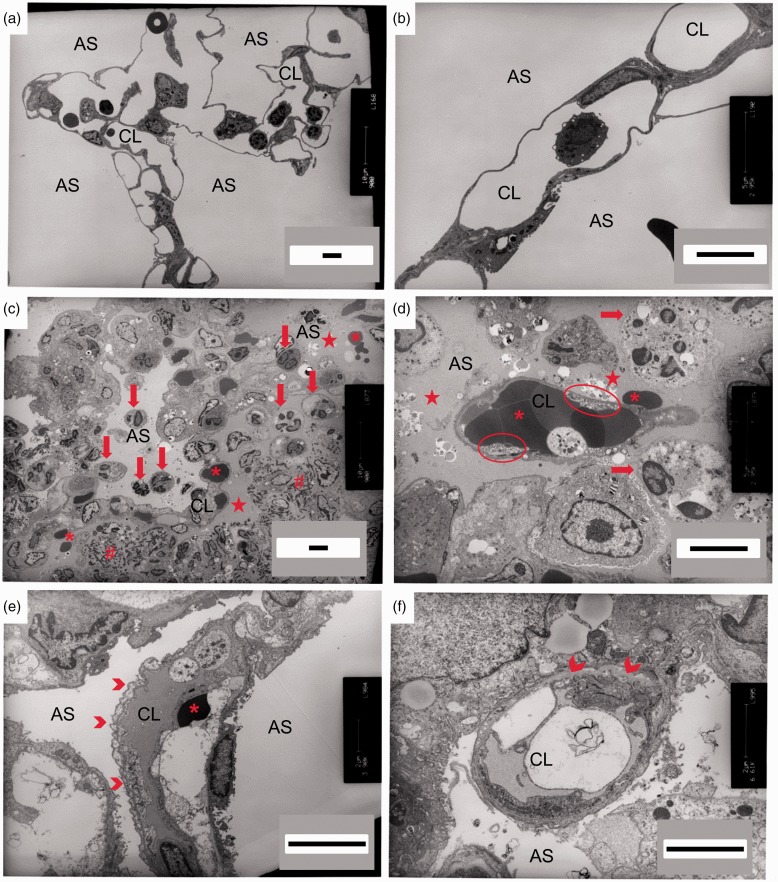
Ultrastructural imaging of distal lung parenchyma during PA103-induced lung injury. Lungs were fixed and sectioned for TEM (reference bar = 5 µm). (a) and (b) Lungs from control animals were characterized by a normal alveolar–capillary structure with thin alveolar walls and clear alveolar spaces. (c) and (d) Lungs from animals at 48 hours post PA103 inoculation were characterized by loss of inter-endothelial junctions with interstitial edema (star), pericapillary edema, and presence of red blood cells (asterisk) and inflammatory cells (arrow). Fibrinoid deposits reminiscent of hyaline membranes were observed in alveolar spaces (hashtag). Microvascular congestion within capillary lumen (Roleaux) and detachment of endothelium from the basal membrane (ellipse) were also evident. (e) and (f) Lungs from animals at 1 week post PA103 inoculation were characterized by basal membrane remodeling and deposition of extracellular matrix (chevron). There was little observable evidence of edema, red blood cells, or inflammatory cell infiltrates in the alveolar space. AS: airspace; CL: capillary lumen.

To more finely resolve the effects of PA103 infection on the pulmonary vasculature during the acute and late phases, we used TEM. Control lungs were characterized by 1) normal alveolar–capillary structure with thin smooth alveolar walls; 2) contiguous endothelial cell junctions; 3) clear alveolar airspaces; and 4) few intravascular and/or lung parenchymal inflammatory cells (Fig. 4(a) and (b)). At 48 hours post PA103 inoculation, lungs were characterized by 1) disrupted alveolar–capillary structure (Fig. 4(c)); 2) loss of inter-endothelial junctions with interstitial edema, pericapillary edema, endothelial blebs, vascular congestion (Roleaux), and endothelial cell sloughing (Fig. 4(d) and S4(a) to (c)); 3) occupancy of airspaces by fluid and fibrinoid deposits (Fig. 4(d) and S4(b)); and 4) intense inflammatory infiltrates within the lung parenchyma (Fig. 4(c) and S4(b)). Of note, Fig. S4(c) and (d) showed evidence of vascular endothelial cells detaching and sloughing from the basal membrane. Lungs from animals at 1 week post PA103 inoculation were characterized by 1) remodeling and deposition of extracellular matrix at the alveolar–capillary barrier (Fig. 4(e) and S5(a) to (d)); 2) contiguous inter-endothelial junctions and hyperplastic endothelial cells around newly formed vessels that were either patent or occupied by extracellular matrix deposits (Fig. 4(e) and (f) and S5A); 3) numerous hyperplastic type II epithelial cells with matrix deposition in some airspaces (Fig. 4(e) and S5(c) to (d)); and 4) few inflammatory infiltrates in the alveolar and interstitial spaces (Fig. S5(b) and (d)). Collectively, these morphologic differences, evidence of matrix deposition, and cellular hyperplasia potentially reflect a disruption and natural resolution of the alveolar–capillary structure.

### Physiological evidence of pulmonary vascular barrier function during P. aeruginosa infection

**Fig. 5.**
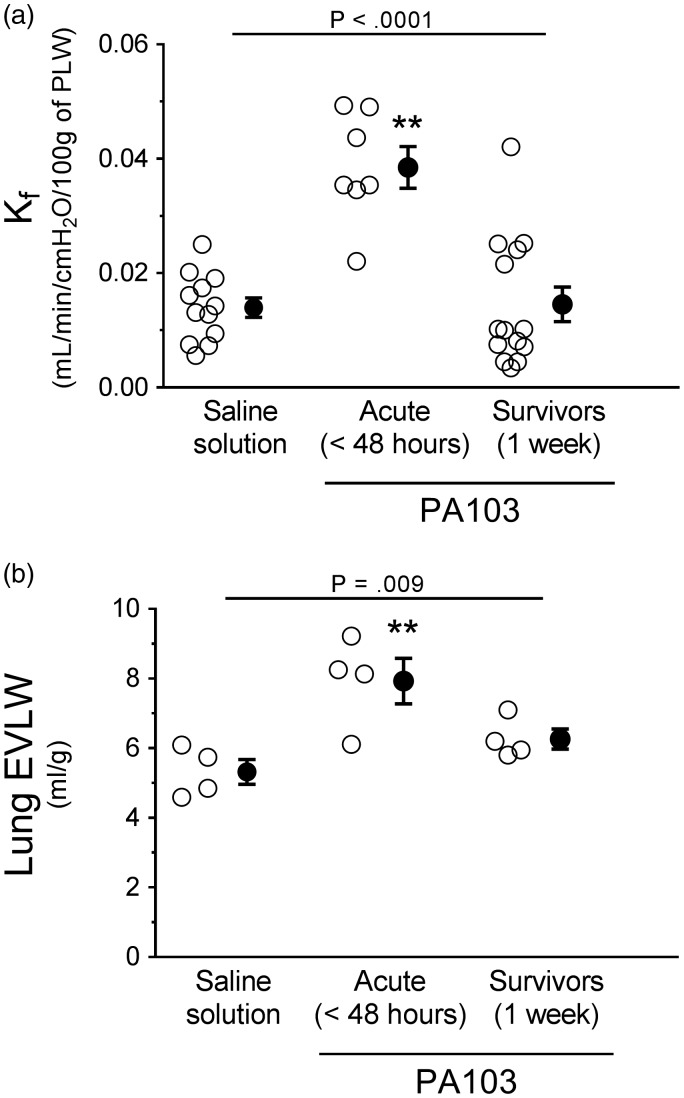
Functional evidence of pulmonary vascular permeability during PA103-induced lung injury. (a) Lungs from control animals (saline solution) or from PA103-inoculated animals (at 48 hours or 1 week post inoculation) were excised *en bloc*, and the filtration coefficient (K_f_) was measured. Compared with controls, lungs from animals at 48 hours post PA103 inoculation displayed a significant increase in K_f_, indicative of pulmonary vascular barrier compromise. Lungs from animals at 1 week post PA103 inoculation were not significantly different from control. **P < .0001 when compared with all other groups by one-way ANOVA. (b) Lungs from control animals (saline solution) or from PA103-inoculated animals (at 48 hours or 1 week post inoculation) were excised and EVLW was measured. Compared with controls, lungs from animals at 48 hours post PA103 inoculation displayed a significant increase in EVLW, indicative of pulmonary vascular barrier compromise. Lungs from animals at 1 week post PA103 inoculation were not significantly different from control animal lungs. **P = .009 when compared with all other groups by one-way ANOVA. Open circles represent measurements from individual lungs and closed circles indicate average ± SEM.

As a sensitive, quantitative, and direct approach to assess pulmonary vascular function, we measured the filtration coefficient (K_f_). In this model, animals were inoculated with saline solution as a control, or inoculated with PA103 as described above. At the indicated time point, the heart and lungs were excised *en bloc*, perfused, and suspended from a force transducer, allowing measurement of lung weight gain. When the pulmonary vasculature is damaged, lungs will gain water weight leaked from the perfusate at a higher rate. Lungs from PA103-infected animals at 48 hours post inoculation displayed significantly increased K_f_ when compared with control or 1-week infected animals, indicating compromise of pulmonary vascular barrier function (Fig. 5(a)). In contrast, K_f_ was not different between controls and 1-week infected animals (Fig. 5(a)). These observed differences in water extravasation into airspaces and interstitium were also reflected by measurements of EVLW (Fig. 5(b)).

### Physiological evidence of gas exchange during P. aeruginosa infection

**Fig. 6.**
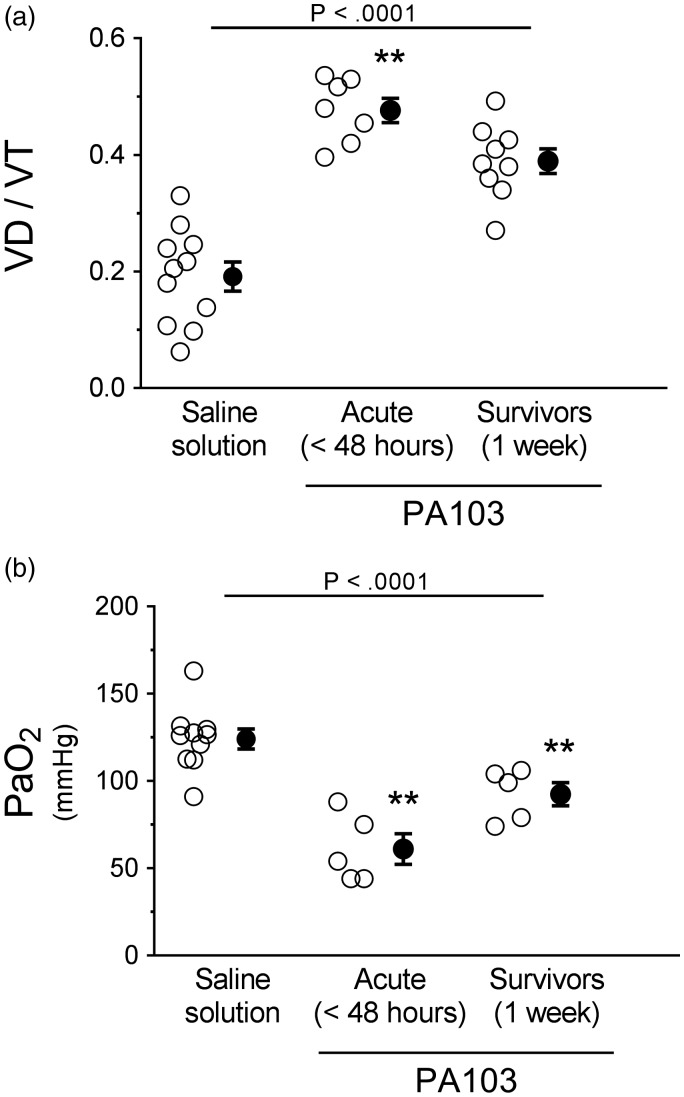
Functional evidence of lung gas exchange during PA103-induced lung injury. Control animals (saline solution), or PA103 inoculated animals (at 48 hours or 1 week post inoculation) were mechanically ventilated to allow measurement of lung gas exchange. (a) VD/VT measures areas of the lung that are ventilated but not perfused. Compared with controls, lungs from animals at 48 hours post PA103 inoculation displayed a significant increase in VD/VT, indicative of pulmonary vascular dysfunction. Lungs from animals at 1 week post PA103 inoculation displayed elevated VD/VT but were not significantly different from control animal lungs. **P < .0001 when compared with all other groups by one-way ANOVA. (b) P_a_O_2_ measures lung gas exchange capability. Compared with controls, lungs from animals at 48 hours and 1 week post PA103 inoculation displayed significant decreases in P_a_O_2_, indicative of impaired gas exchange. **P < .0001 when compared with all other groups by one-way ANOVA. Open circles represent measurements from individual lungs and closed circles indicate average ± SEM.

Considering that airway instillation of PA103 caused edema within injured lung parenchyma, we examined the consequences for gas exchange by measuring VD/VT and P_a_O_2_. The VD/VT was significantly higher at 48 hours post PA103 instillation compared with controls or 1-week infected animals (Fig. 6(a)). The VD/VT in the 1-week infected animals was not significantly different compared with controls (Fig. 6(a)). Compared with controls, the P_a_O_2_ was significantly lower in 48-hour and 1-week infected animals (Fig. 6(b)). There were no differences between 48-hour and 1-week infected animals (Fig. 6(b)). During the exudative phase in this model (48 hours post PA103 instillation), cardiac output was reduced (Fig. S6(a)), while central venous pressure was not altered (Fig. S6(b)), suggesting that the edema from infection was likely non-cardiogenic in nature. Together, these data support the notion that subsequent to epithelial barrier disruption, *P. aeruginosa* infection and/or the attendant endotoxemia/cytokine storm disrupts the pulmonary vascular endothelial barrier function.

### Physiological evidence of lung mechanics during P. aeruginosa infection

**Fig. 7.**
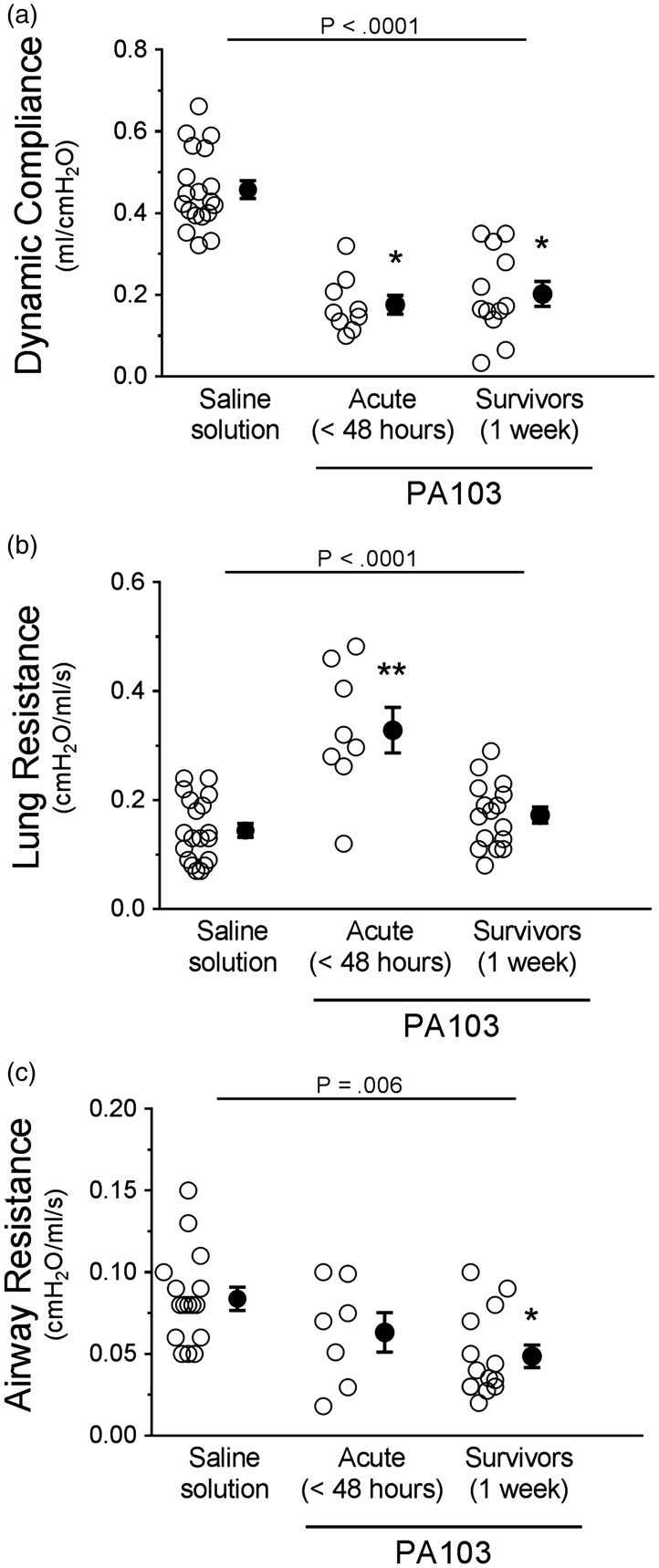
Functional assessment of lung mechanics during PA103-induced lung injury. Control animals (saline solution) and PA103-inoculated animals (at 48 hours or 1 week post inoculation) were mechanically ventilated to allow measurement of lung mechanics. (a) Dynamic compliance measures lung volume for a given applied pressure during periods of air flow. Compared with controls, lungs from animals at 48 hours and 1 week post PA103 inoculation displayed significant decreases in compliance, indicative of lung stiffening due to edema and fibrosis. *P < .0001 when compared with saline solution by one-way ANOVA. (b) Lung airway resistance measures the pressure required to open small airways (i.e., alveolar spaces). Compared with controls, lungs from animals at 48 hours post PA103 inoculation displayed a significant increase in small airway resistance, likely due to decreased dynamic compliance. Lungs from animals at 1 week post PA103 inoculation were not significantly different from control animal lungs. **P < .0001 when compared with all other groups by one-way ANOVA. (c) Airway resistance measures the pressure required to open large airways. Lungs from animals at 48 hours post PA103 inoculation were not significantly different from control animal lungs. Lungs from animals at 1 week post PA103 inoculation were significantly lower compared with control. *P = .006 when compared with saline solution by one-way ANOVA. Open circles represent measurements from individual lungs and closed circles indicate average ± SEM.

To further assess the consequences of pulmonary vascular barrier disruption, we measured multiple parameters of lung compliance. Fluid accumulation in lung airspaces and interstitium, and formation of fibrotic lesions, typically result in lung stiffening along with increased resistance in both small and large airways. Indeed, dynamic compliance (a measure of lung distensibility) was significantly lower at 48 hours and 1 week post PA103 instillation compared with controls (Fig. 7(a)). There were no differences between 48-hour and 1-week infected animals (Fig. 7(a)). Lung resistance was significantly higher at 48 hours post PA103 instillation compared with controls or 1-week infected animals (Fig. 7(b)). The 1-week infected animals were not significantly different compared with controls (Fig. 7(b)). Airway resistance was not significantly different at 48 hours post PA103 instillation compared with controls but was significantly lower at 1 week post PA103 instillation (Fig. 7(c)). The data suggest that changes in dynamic lung compliance are attributed to stiffening of lung parenchyma rather than increases in airway resistance.

**Fig. 8.**
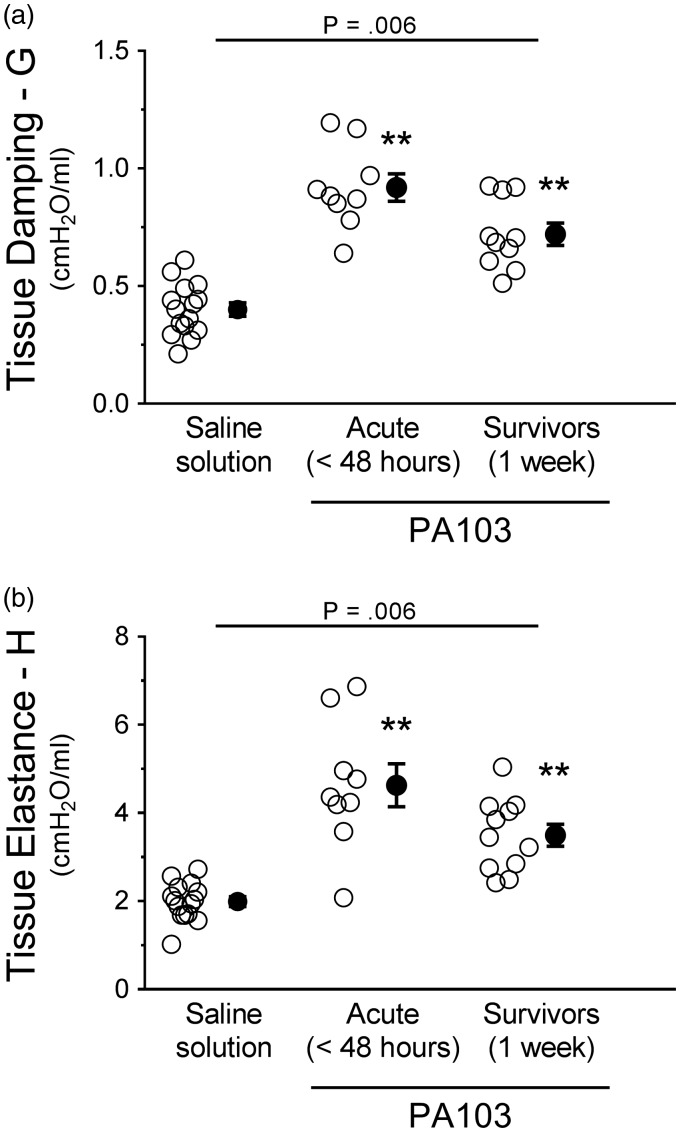
Functional assessment of mechanics in the distal lung parenchyma during PA103-induced lung injury. Control animals (saline solution) or PA103-inoculated animals (at 48 hours or 1 week post inoculation) were mechanically ventilated to allow measurement of mechanics in the distal lung parenchyma. (a) Tissue damping (G) measures the resistive forces of the lung parenchyma, which assesses air flow-independent distal airway elastic recoil. Compared with controls, lungs from animals at 48 hours and 1 week post PA103 inoculation displayed significantly increased tissue damping, indicative of lung stiffening due to edema and fibrosis. **P = .006 when compared with all other groups by one-way ANOVA. (b) Tissue elastance (H) measures the elastic forces of the lung parenchyma, indicating the amount of force needed to overcome the elastic recoil of the lung upon inspiration. Compared with controls, lungs from animals at 48 hours and 1 week post PA103 inoculation displayed significantly increased tissue elastance as a further indication of lung stiffening and fibrosis. **P = .006 when compared with all other groups by one-way ANOVA. Open circles represent measurements from individual lungs and closed circles indicate average ± SEM.

Tissue damping (G) was significantly higher at 48 hours and 1 week post PA103 instillation compared with controls (Fig. 8(a)). There were no differences between 48-hour and 1-week infected animals (Fig. 8(a)). Tissue elastance (H) was also significantly higher at 48 hours and 1 week post PA103 instillation compared with controls (Fig. 8(b)). There were no differences between 48-hour and 1-week infected animals (Fig. 8(b)). Together, these results indicate that disruption of the pulmonary vascular capillary barrier and subsequent accumulation of extravascular fluid within the lung parenchyma and surrounding vessels leads to dysfunction in lung mechanics.

## Discussion

Despite significant advances in critical care and early goal-directed antibiotic therapies, the morbidity and mortality of ARDS remain unacceptably high. Together, this failure and the obvious diversity of ARDS-causing etiologies underscore the possibility that each etiology manifests via unique pathophysiologic mechanism(s) requiring unique intervention(s) beyond the current standard of care. Moreover, ARDS is a continuum between the exudative and reparative/fibro-proliferative phases, which may each require specific interventions. Thus, specific and comprehensively characterized models are needed towards addressing this complex clinical scenario. The present study described the effects of *P. aeruginosa*-induced pneumonia and the resultant progression to ARDS in a rodent infection model.

The exudative phase of ARDS induced by wild type PA103 (48-hour infected animals) was highlighted by morphological and physical evidence of disrupted pulmonary vascular barrier function accompanied by inflammatory cell influx. Examination of the reparative phase (1-week infected animals) revealed an interesting dichotomy between pulmonary vascular barrier function and physiological lung function. The morphological evidence from 1-week infected animals suggested repair of inter-endothelial junctions and fibrinoid deposits within the matrix. This was reflected by quantitative measurements indicating near-normal pulmonary barrier function in 1-week infected animals. However, 1-week infected animals displayed impaired pulmonary compliance, indicative of the lingering effects of infection and inflammation. Thus, despite the potential to contain/clear the infection and repair damaged lung tissue, the persistent effects on physiological lung function suggest that this model may be useful to study long-term ARDS complications.^40–42^ Limitations of the study included a lack of mechanistic insights into the pulmonary vascular endothelial damage, the route of inoculation, the fact that animals are not chronically ventilated concomitantly with infection, the lack of antibiotic treatment to control infection, the fact that animals possessed normal immune systems at the time of infection, the potential for survivor bias, and the descriptive nature of non-quantitative microscopy approaches to assess damage and repair when using small study cohorts.^43^

Acknowledging these limitations, the importance and novelty of this study are underscored by our evaluation of the reparative phase of ARDS, which is not readily assessable in patients. In addition, comparing and contrasting an infectious etiology induced by an ESKAPE pathogen with inflammatory etiologies such as endotoxin insufflation may reveal important differences in the nature of ARDS progression. Thus, future studies will capitalize on this model to extend our understanding of mechanisms underlying repair of the pulmonary capillary endothelium in response to *P. aeruginosa* infection-induced ARDS.

## Acknowledgements

The authors are grateful to Dr. Dan N. Predescu, M.D., and Dr. Sanda A. Predescu, Ph.D. from Rush University in Chicago, Illinois for their interpretation of the transmission electron micrographs included in this manuscript. We thank Dr. Kevin Lowe, M.D., Ph.D., and Ms. Susann Weinholz for their contribution in the initial development of the model. We thank Dr. Jeanine Wiener-Kronish, M.D., for her contribution in experimental design and providing bacterial reagents.

## Conflict of interest

The author(s) declare that there is no conflict of interest.

## Funding

This work was supported by NIH T32 HL076125, AHA 0835134 N, NIH P01 HL066299, and NIH R01 HL118334. The content of this report is solely the responsibility of the authors, who were responsible for study design and execution, and does not necessarily represent the official view of the funding agencies that supported this work.
